# Evaluation of the AIDS clinical trials group staging criteria for Kaposi Sarcoma in a resource limited setting

**DOI:** 10.1186/1750-9378-7-S1-P8

**Published:** 2012-04-19

**Authors:** Fred Okuku, Jackson Orem, James Kafeero, Warren Phipps, Moses R Kamya, Corey Casper

**Affiliations:** 1 Uganda Cancer Institute, Kampala, Uganda; 2 Department of Medicine, Makerere University, College of Health Sciences, Kampala, Uganda; 3 Department of Medicine, University of Washington, Seattle, WA, USA; 4 Laboratory Medicine, University of Washington, Seattle, WA, USA; 5 Epidemiology, University of Washington, Seattle, WA, USA; 6 Global Health, University of Washington, Seattle, WA, USA; 7 Vaccine and Infectious Disease Division, Fred Hutchinson Cancer Research Center, Seattle, WA, USA

## Background

Kaposi sarcoma (KS) is commonly staged using by the AIDS Clinical Trials Group (ACTG) criteria. The three variables of the ACTG are dichotomized as good risk (0) and poor risk (1). Good risk immune status (I0) is defined as CD4 T-cell count ≥200cells/µl, and poor risk (I1) as CD4 < 200cells/µl. Although validated in the US and Europe, no evaluation has been done in resource-limited settings during the HAART era. We sought to determine whether the ACTG staging criteria is predictive of overall survival among Ugandan patients with HIV-associated KS.

## Methods

Data were abstracted from medical records of adult patients with HIV-associated KS seen at the Uganda Cancer Institute (UCI) from 2000-2006. The primary outcome was 2-year overall survival. Vital status at 2 years was determined from the medical chart, or by contacting the patient or next of kin using the phone contact provided in the chart or ART clinic. Survival was modeled using Kaplan-Meier methods. Factors associated with survival were evaluated using Cox proportional hazards.

## Results

The median survival time was 468 days (range 0, 5411). At 2 years following KS diagnosis, 165 (40.8%) of participants were alive and 166 (41.1%) had died, while 73(18.1%) were lost to follow-up. Factors associated with death before 2 years from KS diagnosis included T1 tumor stage, S1 stage, nodular lesion morphotype, and trunk edema (Table 1). Baseline CD4 count under 100 cells/µl was associated with decreased survival (HR 1.7, 95%CI 1.26-2.39 and p= 0.001), but ACTG immune status criteria (CD4 under 200 cells/µl) was not.

**Table 1:** Factors associated with death before 2 years from KS diagnosis

	Univariate			Multivariate		
**FACTOR**	**HR**	**95%**	**CI P-value**	**HR**	**95%**	**CI P-value**

T1 VS T0	4.13	2.18-7.81	<0.001	4.33	2.36-8.77	<0.001
I1 VS I0	1.25	0.64-2.44	0.52	……..	…………	………..
S1 VS S0	1.71	1.13-2.57	0.01	1.69	1.12-2.53	<0.01
Age (yrs)	0.98	0.96-1.00	0.05	0.98	0.96-1.00	0.01
Nodular KS morphotype	1.50	0.97-2.32	0.07	1.34	0.84-2.15	0.22
Trunk edema	2.91	1.53-5.53	<0.001	2.45	1.27-4.75	0.01
On HAART at diagnosis	0.75	0.54-1.02	0.07	0.62	0.45-0.87	0.01
Receipt of chemotherapy	0.46	0.33-0.65	<0.001	0.29	0.20-0.42	<0.001

* Multivariate analysis adjusted for T, S, age, nodular morphotype, trunk edema, HAART, and chemotherapy.

## Conclusions

ACTG criteria Tumor extent (T) and Systemic symptoms (S) were associated with survival; Immune status (I) was not. Factors associated with decreased survival included: baseline CD4 counts <100, age, trunk edema, while receipt of HAART and chemotherapy were associated with increased survival. Studies are needed to validate ACTG staging criteria in sub-Saharan Africa and to identify additional prognostic factors.

